# The Developmental Disorders of Fall Armyworm (*Spodoptera frugiperda*, Lepidoptera: Noctuidae) Caused by the Infection with *Nosema* sp. (Microsporidia: Nosematidae)

**DOI:** 10.3390/microorganisms13050994

**Published:** 2025-04-26

**Authors:** Yudi Xu, Haoyu Liu, Xinzheng Huang, Shuqian Tan, Wangpeng Shi

**Affiliations:** MOA Key Lab of Pest Monitoring and Green Management, Department of Entomology, China Agricultural University, Beijing 100193, China; 15372123367@163.com (Y.X.); 2021319010407@cau.edu.cn (H.L.);

**Keywords:** *Spodoptera frugiperda*, *Nosema*, pathology, insect development, transcriptome, chitin synthesis

## Abstract

The fall armyworm (*Spodoptera frugiperda*), a globally invasive pest, poses substantial threats to corn in China. Microsporidia are a group of obligate intracellular parasitic fungi and are considered to have great potential in biological control. In this article, we investigated the pathology of *Nosema* sp. infection in *S. frugiperda* larvae at the organismal, cellular, and molecular levels. At the organism level, this microsporidian significantly prolonged the developmental duration of the host, reduced its body weight, caused molting failure, and led to a high mortality rate at 98.9%, 97.8%, and 64.0%, respectively, in 5 × 10^5^, 5 × 10^4^, 5 × 10^3^ spores/larva doses. Microsporidia infection caused severe damage to midgut cells, including the formation of vacuoles in the cytoplasm, mitochondria, and intercellular spaces, destruction of goblet cells, and partial encapsulation of spores by mitochondria. Transcriptomic profiling revealed significant alterations in gene expression profiles in *S. frugiperda* larvae following microsporidian infection. The expression levels of genes associated with the chitin synthesis pathway (*CHS1*, *G6PI*, *GFAT*, *GNPNA*, *PAGM*, *UAP*) were inhibited, which may contribute to the effects of *Nosema* sp. on the growth and development of *S. frugiperda*.

## 1. Introduction

The fall armyworm (*Spodoptera frugiperda*), a globally distributed agricultural pest originating from the Americas, has posed severe threats to China’s food security since its invasion in 2019 [1]. Characterized by strong migratory capacity, high fecundity, broad host range, and voracious feeding habits, this pest has caused significant damage to China’s corn production and has been classified as a Class I crop pest in China [2,3]. While chemical control remains effective in managing fall armyworm infestations, it carries the risk of inducing pesticide resistance [4]. In contrast, biological control offers distinct advantages including environmentally friendly, long-term effectiveness, and low resistance risk. Recent advances in biological control strategies against fall armyworms have been achieved through the application of various natural enemies and pathogens, including parasitic wasps, *Beauveria bassiana*, *Metarhizium anisopliae*, *Bacillus thuringiensis*, nucleopolyhedroviruses (NPVs), and microsporidia [5,6,7,8].

Microsporidia are obligate intracellular parasitic fungi ubiquitously distributed across nearly all vertebrates and invertebrates, particularly insects [9]. Entomopathogenic microsporidian species are considered promising agents for biological control [10]. Microsporidia infections in insects could cause multi-level pathological effects. At the organismal level, infected individuals exhibit altered color, developmental stunting, morphological aberrations, and behavioral modifications compared to healthy counterparts [8,10,11,12]. At the cellular level, infection induces cytopathological effects, including cell and nuclear hypertrophy, vacuolation, xenomas, and apoptosis dysregulation [10,13,14]. At the molecular level, microsporidian parasitism triggers extensive transcriptomic reprogramming, significantly altering host gene expression profiles [15]; for example, infection of *Bombyx mori* by *Nosema bombycis* alters expression of genes governing juvenile hormone (JH) biosynthesis and immune pathways, critical regulators of silkworm development [16]. The genes that regulate the cell cycle are modulated when bees are infected by microsporidia [17]. *Tribolium castaneum* infected by *Paranosema whitei*, the chitin metabolic genes changed [18].

As a fundamental structural biopolymer, chitin serves critical roles in insect exoskeletons, tracheal systems, internal tendon structures, and intestinal peritrophic matrices [19]. Its biosynthesis pathway involves eight key enzymatic steps mediated sequentially by: trehalase (TRE), hexokinase (HK), glucose-6-phosphate isomerase (G6PI), glutamine-fructose-6-phosphate aminotransferase (GFAT), glucosamine-6-phosphate N-acetyltransferase (GNPNA), phosphoacetylglucosamine mutase (PAGM), UDP-N-acetylglucosamine pyrophosphorylase (UAP), and chitin synthase (CHS) [20]. There are two types of chitin synthases in most insects. Among them, *CHS1* is mainly present in the cuticle and trachea, while *CHS2* is present in the peritrophic membrane. CHS plays an important role in growth and development, molting, and pupation [19]. *CHS1* is regulated by various factors, including hormones (e.g., 20E), cuticle damage, carbohydrate metabolism, miRNAs, and chitin synthesis inhibitors [21,22,23]. The infection of the host by microsporidia can have an impact on chitin metabolism [18], but there is currently no report on its effect on chitin synthesis.

In this article, we evaluated developmental impacts on fall armyworm larvae infected by *Nosema* sp., and ultrastructural histopathological analysis was exhibited. At the molecular level, transcriptomic profiling coupled with targeted validation identified significant regulatory alterations in chitin synthesis pathways.

## 2. Materials and Methods

### 2.1. Insects and Microsporidia

The fall armyworm larvae were bought from Henan Jiyuan Baiyun Industry Co., Ltd. (Jiyuan, China) and kept in insect rearing room (27 ± 2 °C, 60 ± 5% humidity and 14:10 photoperiod) for at least 5 generations. For spore production, 4th instar larvae were starved for 6–8 h and fed with artificial diets (2 mm × 2 mm × 2 mm) polluted by Microsporidia. After the diets were consumed, the larvae were fed with clean diets. The dead larvae were collected and homogenized in sterilized distilled water, and the homogenate was filtered through absorbent gauze. The spore filtrate was purified by 90% Percoll gradient centrifugation, the spore pellet was resuspended in sterilized distilled water and stored at 4 °C until further use [24].

### 2.2. Latent Period Detect

Three concentrations (1 × 10^9^, 1 × 10^8^, 1 × 10^7^ spores/mL) were prepared, every group infected 15 fourth instar larvae with 0.5 μL spore suspension (5 × 10^5^, 5 × 10^4^, 5 × 10^3^ spores/larva) as describe above and each larva were kept in a 25 mL plastic cup. The cup and diet were changed daily, and frass was collected daily to check whether the microsporidia present under 400× light microscopy by fresh wet smears. The period from feeding spores to the first appearance of spores in frass is latent period [10,25]. The data were found to follow a normal distribution by the Shapiro–Wilk test. One-way ANOVA analysis and Tukey’s multiple comparisons test were used to check significance.

### 2.3. Ultra-Histopathology

Starved fourth instar larvae were inoculated with 5 × 10^5^ spore/larva; after 4 days, infected and healthy midgut tissues were dissected and immediately fixed in 2.5% glutaraldehyde (pH 7.4) at 4 °C for 24 h. Tissues were rinsed in 0.2 M phosphate buffer and post-fixed in 1% osmium tetroxide (OsO_4_ in 0.2 M phosphate buffer) for 2 h at 4 °C, and dehydrated in 30%, 50%, 60%, 70%, 80%, 90%, 95%,100% ethanol, then embedded in epoxy resin. Ultrathin sections (80–100 nm) were taken with Leica EM UC7 (Leica Microsystems, Wetzlar, Germany), and stained with saturated uranyl acetate and Reynolds’ lead citrate. Samples were observed under a HITACHI HT7800 (Hitachi High-Tech Corporation, Tokyo, Japan) transmission electron microscope.

### 2.4. Impact of Nosema sp. on Development of S. frugiperda

Starved fourth instar larvae were inoculated with 5 × 10^5^, 5 × 10^4^, 5 × 10^3^, and 0 spores/larva as described above; each treatment group was set up with 3 replicates and 30 individuals per replicate. The larvae were checked to determine whether dead twice a day, and the age of death and developmental duration of each instar were recorded. In each group, 30 larvae were randomly selected, and their body weights were recorded daily until death or until the 10th day (whichever occurred first). The data were found to follow a normal distribution by the Shapiro–Wilk test. One-way ANOVA analysis and Tukey’s multiple comparisons test were used to check significance.

### 2.5. RNA-Seq

Starved fourth instar larvae were inoculated with 5 × 10^5^ spore/larva; after 4 days (24 h of 5th instar), total RNAs were extracted from healthy and infected larvae for constructing RNA sequencing libraries, and each treatment group was set up with 3 replicates and 3 individuals per replicate. The sequencing libraries were sequenced on the Illumina HiSeq 2000 platform by Beijing Tsingke Biotechnology Co., Ltd. (Beijing, China). Transcripts were annotated on the basis of the reference genome (http://v2.insectgenome.com/Organism/715, accessed on 3 April 2024). DESeq2 was used to analyze the differential expression of identified genes [26], and those with *p* < 0.05 and fold change ≥2 were considered to be significantly differentially expressed. Gene ontology (GO) enrichment and KEGG pathway analysis (https://www.genome.jp/kegg/, accessed on 10 April 2024) were used to identify the functional modules of differential expression genes.

### 2.6. Impact of Nosema sp. on Genes of Chitin Synthesis Pathway of S. frugiperda

Starved fourth instar larvae were inoculated with 5 × 10^5^ spores/larva as described above. The larva at 12 h, 24 h, and 48 h of the fifth instar were collected, and total RNA was extracted by Trizol (TRIzol^®^ Reagent, Ambion^®^, Thermo Fisher Scientific Inc., Waltham, MA, USA). Three larvae were taken at each time point, and three replicates were set up. The RNA quality and concentration were measured with a micro nucleic acid analyzer NanoDrop™ 2000 (Thermo Fisher Scientific Inc., Waltham, MA, USA). A total of 1 μg of RNA was reverse transcribed into cDNA according to instruction (TRUE script RT MasterMix, Aidlab Biotechnologies Co., Ltd., Beijing, China). The gene expression was detected with M5 HiPer SYBR Premix EsTaq plus (with Tli RNaseH) kit (Mei5 Biotechnology Co., Ltd., Beijing, China) in a Bio-Rad CFX Connect Real-Time System (Bio-Rad Laboratories Inc., Hercules, CA, USA). qPCR system: M5 HiPer SYBR Premix EsTaq plus 10 μL, forward primer 0.5 μL, reverse primer 0.5 μL, cDNA 1 μL, DNase/RNase-free water 8 μL. qPCR procedure: 95 °C for 30 s; 40 cycles of 95 °C for 5 s and 60 °C for 40 s; 95 °C for 10 s; melt curve at 60–95 °C. The genes related to chitin synthesis and housekeeping gene primers are shown in Table S1. Three biological and three technical replicates were set up. Moreover, 2^−ΔΔCT^ method was used for data processing [27], and *t*-test was used for significance analysis. The data of the Infected group and the Control group were each put through the Shapiro–Wilk normality test, one-way ANOVA, and Tukey’s multiple comparisons test.

## 3. Results

### 3.1. Ultra-Histopathology Within Latent Period in S. frugiperda Infected by Nosema sp.

The periods were 6.53 ± 1.60 (*n* = 15), 8.00 ± 1.85 (*n* = 15), and 9.50 ± 1.51 (*n* = 10) (mean ± SD) days in 5 × 10^5^, 5 × 10^4^, and 5 × 10^3^ spores/larva groups, respectively. One-way ANOVA analysis showed that inoculation dose significantly affected the latent period (F _2,37_ = 9.5, *p* < 0.05), and there was a significant difference between 5 × 10^5^ and 5 × 10^3^ spores/larva groups (*p* = 0.0003) (Figure S1)

Ultrastructural analysis of midgut tissues from *S. frugiperda* larvae at 4 days post-microsporidian inoculation revealed distinct pathological alterations (Figure S2). The most prominent of these was the appearance of vacuoles within the cytoplasm located in close proximity to the mitochondria. And the mitochondria became thinner and longer, while the normal mitochondria are oval in shape. The mitochondria were observed to semienclose the spores, and it is hypothesized that this might be the reason for the elongation of the mitochondria. Vacuoles were also present in the intercellular spaces and mitochondria. Another obvious histological lesion was the detachment of goblet cells, and microvilli broke and filled the goblet cavity, while in normal circumstances, goblet cells adhere closely to columnar cells.

### 3.2. Impact of Developmental Duration, Weights, and Virulence on S. frugiperda Infected by Nosema sp.

**Figure 1:**
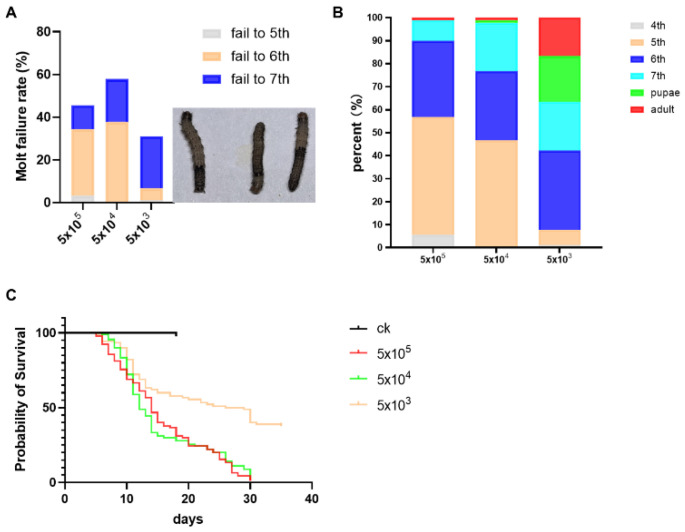
(**A**): The molt failure rate in different instar stages and the symptom of molt failure; (**B**): the proportion of deaths at different instar stages after the 4th instar larvae were infected by *Nosema* sp.; (**C**): survival curves of S. frugiperda infected by *Nosema* sp. at different dosages.

The symptom of molting failure was identified as the primary cause of mortality, and the molt failure rates were 45.6%, 57.8%, and 31.5% in 5 × 10^5^, 5 × 10^4^, and 5 × 10^3^ spores/larva groups, respectively (Figure 1A). Under different inoculation doses, the lethal age of microsporidia to *S. frugiperda* larvae was different. At the dose of 5 × 10^5^ spores, a few individuals died at the 4th instar, and the larvae mainly died at the 5th and 6th instars. At the dose of 5 × 10^4^ spores, individuals mainly died at the 5th, 6th, and 7th instars. And at the dose of 5 × 10^3^ spores, the 6^th^ and 7th larvae and pupae died mainly (Figure 1B). And the mortality in the larvae stage was 98.9%, 97.8%, and 64.0%, respectively, from high to low dose, and the median lethal time (LT_50_) was calculated to be approximately 14, 12, and 27.5 days, respectively, from high to low dose (Figure 1C).

**Figure 2:**
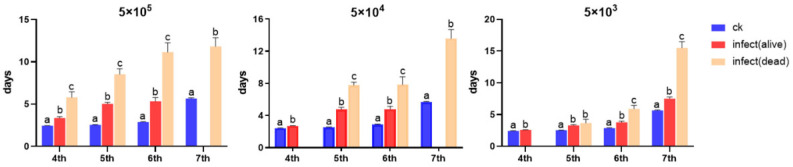
The developmental duration of *S. frugiperda* larvae under different inoculation doses of *Nosema* sp. Letters represent the results of One-way ANOVA analysis and Tukey’s multiple comparisons among uninfected individuals, infected surviving individuals, and infected dead individuals at the same instar stage (*p* < 0.05). The absence of columns represents that *n* < 3, which is not included in the statistics.

The developmental duration is shown in Table 1. Larvae exhibited significantly prolonged developmental duration across all instars following microsporidia inoculation. Notably, infected larvae that succumbed during each respective instar showed significantly extended survival periods compared to their surviving individuals, except at the 5th instar infected with 5 × 10^3^ spores (Figure 2).

**Figure 3:**
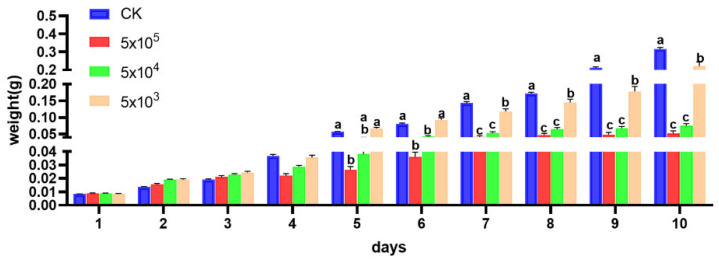
The daily body weight of *S. frugiperda* after being infected by *Nosema* sp. Letters represent the results of One-way ANOVA and Tukey’s multiple comparisons among different treatment groups at the same time point (*p* < 0.05).

The daily body weights of larvae were measured within 10 days after microsporidia infection when larvae were 4th instar. The results showed that within 4 days post-infection, there was no significant change in body weight among the treatment groups. On the 5th day, a significant difference emerged between the 5 × 10^5^ group and the control group (*p* = 0.0004). On the 6th day, a significant difference was observed between the 5 × 10^4^ group and the control group (*p* < 0.0001). On the 7th day, a significant difference occurred between the 5 × 10^3^ group and the control group (*p* = 0.0059). Notably, there was no difference between the 5 × 10^5^ group and the 5 × 10^4^ group throughout the experiment, while a significant difference persisted between the 5 × 10^5^ group and the 5 × 10^3^ group starting from the 5th day. These findings indicate that microsporidia infection significantly affects the body weight of the host, and different inoculation doses also have a significant impact on the host’s body weight (Figure 3).

### 3.3. RNA-Seq Analysis

**Figure 4:**
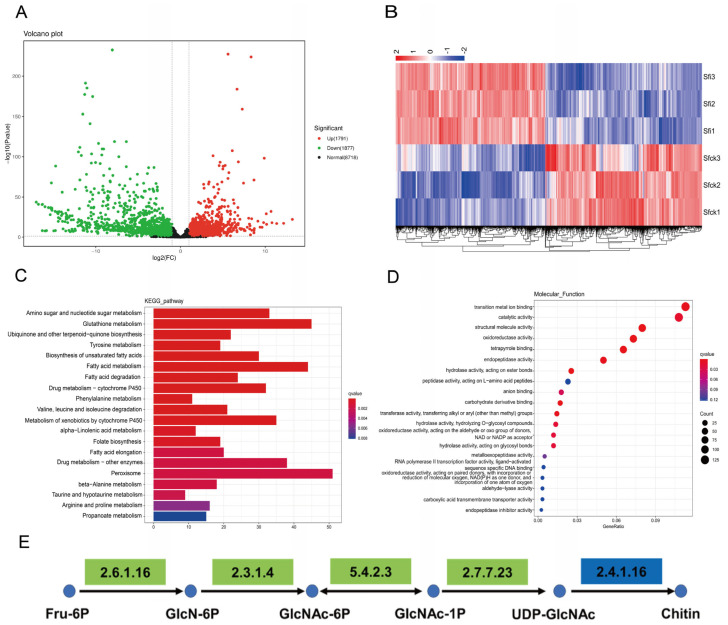
Transcriptome analysis of DEGs between the healthy and *Nosema* sp. infected *S. frugiperda*. (**A**): Volcano plots showing the DEGs between the healthy and *Nosema* sp. infected *S. frugiperda*, red dots represent upregulated genes in infected group, green dots represent downregulated genes in infected group, and black dots indicate genes with no significant differences. (**B**): Heatmap of DEGs. Blue represents a lower level of expression, while red represents a higher level of expression. (**C**): KEGG pathway enrichment. (**D**): GO enrichment (Molecular Function). (**E**): KEGG map. Chitin synthesis pathway (Starting from Fru-6P, they are GlcN-6P, GlcNAc-6P, GlcNAc-1P, UDP-GlcNAc, and Chitin in sequence) in Amino sugar and nucleotide sugar metabolism pathway (ko00520). Green represents downregulated genes, and blue represents the genes upregulate and downregulate.

The main sequencing information of RNA-seq is shown in Table S2. Transcriptome analysis results indicated that *S. frugiperda* following microsporidian infection identified 3668 differentially expressed genes (DEGs), comprising 1791 upregulated and 1877 downregulated transcripts (Figure 4A). KEGG enrichment analysis revealed that the Amino sugar and nucleotide sugar metabolism pathway was one of the most significantly enriched pathways (Figure 4C). GO analysis demonstrated that the differentially expressed genes were predominantly concentrated in the category of Catalytic activity (Figure 4D). Notably, we found that genes encoding relevant enzymes in the hexosamine pathway of the chitin synthesis pathway were annotated in both the Amino sugar and nucleotide sugar metabolism pathway and the Catalytic activity category. Among these genes, *GFAT* (EC 2.6.1.16), *GNPNA* (EC 2.3.1.4), *PAGM* (EC 5.4.2.3), and *UAP* (EC 2.7.7.23) exhibited consistent downregulation, and *CHS* (EC 2.4.1.16) displayed bidirectional regulation (Figure 4E).

### 3.4. Gene Expression of Chitin Synthesis Pathway

**Figure 5:**
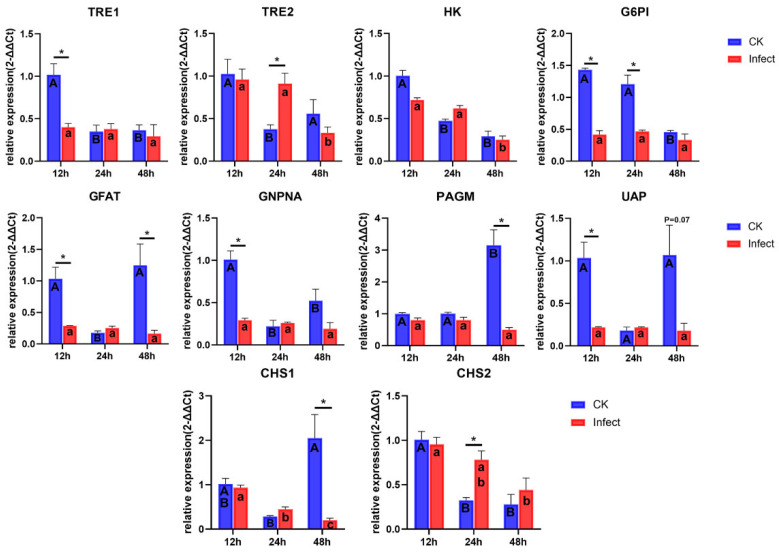
Gene expression of chitin synthesis in 5th instar *S. frugiperda* at different times. The bar showed SE, “*” represents significant differences between CK and Infect group (*t*-test, *p* < 0.05). Uppercase and lowercase letters represent significant temporal variations in gene expression within control and infected groups, respectively (One-way ANOVA Analysis and Tukey’s multiple comparisons, *p* < 0.05).

The expression levels of all genes in the chitin synthesis pathway at three different time points in the 5th instar larvae of *S. frugiperda* were measured (Figure 5). Compared to uninfected counterparts, infected larvae exhibited significantly reduced expression of *TRE1*, *G6PI*, *GFAT*, *GNPNA*, and *UAP* genes at 12 h of 5th instar larvae. At 24 h, *G6PI* gene expression was significantly reduced in infected larvae, while *TRE2* and *CHS2* genes were significantly upregulated. And at 48 h post of 5th instar, infected larvae had significantly lower expression of *GFAT*, *PAGM*, and *CHS1* genes. These results were generally consistent with the transcriptome analysis results. Additionally, significant temporal dynamics were observed in the expression of chitin synthesis pathway genes in uninfected larvae, but some genes in infected individuals showed no significant variations at different times (e.g., *TRE1*, *G6PI*, *GFAT*, *GNPNA*, *PAGM*). Notably, the expression of *CHS1* decreased significantly over time, indicating that microsporidian infection may affect the expression levels of genes in the chitin synthesis pathway, ultimately disrupting the normal process of chitin synthesis. We hypothesize that this disruption contributes to the prolonged developmental duration and molting failure of infected *S. frugiperda* larvae.

## 4. Discussion

The latent period of a pathogen is the time between the invasion of the host and the first production of infectious units [28] or the time between the invasion of the host and the first appearance of symptoms [29]. In this article, the latent period of microsporidia ranged from 6 to 10 days, depending on inoculation doses. Symptom manifestation (e.g., weight loss and prolonged developmental duration) preceded the detection of infective spores. This phenomenon occurs because newly generated microsporidian spores do not immediately release into the environment post-host infection but instead continue propagating within the host body [10]. Consequently, pathological damage has already commenced during the latent period of microsporidian infection.

Following microsporidian infection in insects, particularly lepidopteran hosts, the midgut emerges as the primary target tissue. To enable direct visualization of cellular pathologies induced by microsporidian infection, we employed transmission electron microscopy (TEM) for histopathological analysis. The infection of the host by *Nosema* sp. within the latent period had already caused severe damage to the cells. The most obvious symptom observed was cytoplasmic vacuolization, a phenomenon previously documented in *Nosema*-infected hosts across multiple studies; however, this feature is not microsporidian-specific, as similar vacuolization patterns occur during *Bacillus thuringiensis* (Bt) infections [13,30]. Vacuoles appear around the mitochondria, and this phenomenon has also been confirmed in cells infected by *Encephalitozoon* microsporidia [31,32]. We have also observed some new pathological phenomena, such as the formation of vacuoles within mitochondria and the direct encapsulation of spores by mitochondria. Microsporidia lack mitochondria and need to obtain energy directly from the host [33]. The interaction between microsporidia and the host mitochondria that we have observed indicates that the energy source of microsporidia is the host mitochondria. The infection of host cells by *Nosema* sp. can also cause damage to goblet cells, such as the microvilli break; this can compromise ionic homeostasis and nutrient absorption, impairing physiological mechanisms of detoxification [34]. The insect midgut regulates growth and development through coordinated regulation of nutrient metabolism, hormonal signaling, and gut microbiota dynamics [35,36,37]. Therefore, *Nosema* sp. may inhibit the growth and development of the host by damaging the intestinal tissues.

Microsporidian infections are classified as chronic (slow-acting, progressing seriousness) or acute (fast-acting, short duration), depending on the microsporidian species, inoculation dose, and host instar at infection [10,12,38]. The latent period of microsporidian infection in 4th instar *S. frugiperda* larvae (approximately 5–9 days) was relatively long compared to the normal developmental duration from the 4th larval stage to pupation (approximately 12 days). And the characteristic manifestations of developmental retardation and gradual mortality post-infection align with the definition of chronic infection. However, we concurrently observed accelerated mortality in a minority of infected individuals, which may be attributed to compromised host immune competence. Thus, the manifestation of microsporidian infection in *S. frugiperda* as either chronic or acute infection is contingent upon the host’s physiological condition.

Chitin, an essential biopolymer in insects absent in plants and vertebrates, represents an ideal target for pest control strategies. Chitin synthase (CHS), the pivotal enzyme in chitin biosynthesis, plays a critical role in insect development [19]. In *S. frugiperda*, *CHS1* exhibits predominant expression in the epidermis, and its silencing via SfCHS1-siRNA nanocomplex disrupts larval molting and pupation [39]. The chitin synthesis inhibitor 2,6-difluorobenzoylurea inhibits the expressions of *TRE1*, *HK*, *G6PI*, *GFAT*, and *CHS1*, resulting in the obstruction of molting in *S. frugiperda* and ultimately leading to its death [40]. Transcriptome analysis revealed widespread suppression of genes within the chitin biosynthesis pathway. qPCR validation demonstrated significant *CHS1* downregulation in microsporidia-infected larvae at 48 h post-5th-instar. Concurrently, healthy larvae initiated molting preparations while infected counterparts exhibited complete molting arrest, suggesting microsporidia prolong larval development through targeted *CHS1* suppression, thereby blocking ecdysis progression.

Notably, similar to the results of the transcriptome analysis, the expression level of *CHS2* in the infected larvae was upregulated at 24 h post-5th instar. As *CHS2* primarily functions in midgut peritrophic membrane formation [19], and our ultrastructural histopathology confirmed microsporidia-induced midgut cell damage, we speculate that the observed *CHS2* upregulation may reflect a compensatory mechanism to repair peritrophic membrane lesions caused by infection, this hypothesis warrants further experimental validation. The expression pattern of *TRE2* is similar to that of *CHS2*, indicating that *TRE2* is related to the peritrophic membrane in *S. frugiperda*. The expression levels of other genes in the chitin synthesis pathway (including *TRE1*, *G6PI*, *GFAT*, *GNPNA*, *PAGM*, and *UAP*) were also inhibited after *S. frugiperda was* infected by *Nosema* sp. The inhibition of upstream genes in the chitin synthesis pathway also affects the growth and development of the host [41,42]. In addition, our results demonstrated genes of the chitin synthesis pathway exhibited dynamic expression patterns during normal larval development, but after microsporidia infection, the expression levels remained stable or were continuously inhibited, which may be one of the reasons for host developmental obstruction.

## Figures and Tables

**Table 1:** The developmental durations (days) of surviving and dead individuals at each instar stage in 4th instar larvae under different inoculation doses.

	ck	5 × 10^5^		5 × 10^4^		5 × 10^3^	
Instar	Alive	Alive	Dead	Alive	Dead	Alive	Dead
4th	2.44 ± 0.32 *n* = 90	3.36 ± 1.53 *n* = 85	5.80 ± 1.48 *n* = 5	2.70 ± 0.35 *n* = 90	/	2.58 ± 0.23 *n* = 89	/
5th	2.54 ± 0.40 *n* = 90	5.03 ± 1.20 *n* = 38	8.53 ± 4.39 *n* = 47	4.75 ± 1.85 *n* = 48	7.77 ± 2.50 *n* = 42	3.31 ± 1.03 *n* = 83	3.67 ± 1.50 *n* = 6
6th	2.88 ± 0.50 *n* = 90	5.33 ± 1.35 *n* = 9	11.16 ± 5.93 *n* = 29	4.80 ± 1.50 *n* = 20	7.85 ± 5.00 *n* = 27	3.77 ± 1.38 *n* = 52	5.90 ± 3.05 *n* = 31
7th	5.66 ± 0.80 *n* = 88	/	11.81 ± 2.95 *n* = 9	/	13.58 ± 4.83 *n* = 25	7.53 ± 1.51 *n* = 33	15.50 ± 4.29 *n* = 19

Mean ± SD, “/” indicates that *n* < 3, and these data are not included in the statistics.

## Data Availability

The original contributions presented in this study are included in the article/Supplementary Material. Further inquiries can be directed to the corresponding authors.

## Supplementary Materials

The following supporting information can be downloaded at: https://www.mdpi.com/article/10.3390/microorganisms13050994/s1. Table S1: The primers for qPCR; Table S2: Sequencing information of RNA-seq; Figure S1: Latent period in *S. frugiperda* infected by *Nosema* sp.; Figure S2: Ultra-histopathology of the midgut of *S. frugiperda* infected by *Nosema* sp.
