# Supplementary material for: The Developmental Disorders of Fall Armyworm (Spodoptera frugiperda, Lepidoptera: Noctuidae) Caused by the Infection with Nosema sp. (Microsporidia: Nosematidae)
Source: Microorganisms. 2025 Apr 26;13(5):994. doi: 10.3390/microorganisms13050994 (PMC12114601; doi:10.3390/microorganisms13050994)
Supplement: Supplementary file 1 [file microorganisms-13-00994-s001.zip › microorganisms-3543452-supplementary.pdf]

Table S1. The primers for qPCR.

Gene	primer (5'-3')
SfTRE1	TCAGATGAAGGTGAACTCGAAGA
	GGAATGATGAATCCGTGGGTA
SfTRE2	CTGCTGCTGTCGGAGATGA
	TAGGAGGGGAGGCTGTGAT
SfHK	TTATGACTGGTACAGGTGTCGCC
	ATCCCCTCGTCTAGCAATCG
SfG6PI	CAGTGGGCAGTGGAAAGGG
	TCAGTTGGTTGGCGTAGGGT
SfGFAT	GTGCCCTCTGTTCCCCG
	TGCTGCTGCCCGAGAGA
SfGNPNA	ATCCTCCAGAGATACTCAAGCGT
	ATATACGGGTGTCCTCGATCACT
SfPAGM	TCTTGTTAGAACATGTGGTCTACCG
	CCTCCAGCATTTCACCGTCA
SfUAP	CAGTGTTCGCTTTCTAATCGCC
	TTCTTGAATAACTTTGCTCGTATCG
SfCHS1	ACGTTGGTCTCAGGTGATGT
	GCAGCCAATGACCAATAGCG
SfCHS2	GAATTTAGGAGCAGCGTGCG
	GCAGCCAATGACCAATAGCG
SfActin	GATCTGGCACCACACCTTCT
	GGCGTGTTGAAGGTCTCGAA
SfGADPH	CCGTTGACATGCAAGATGGC
	AGACGCCTTCTCTGTGGTTG

Table S2. Sequencing information of RNA-seq.

Samples	Clean reads	GC Content	% ≥ Q30	Mapped reads
ck1	20,172,416	47.09%	90.64%	77.84%
ck2	18,070,756	47.13%	89.79%	77.29%
ck3	21,290,429	47.68%	90.99%	78.65%
infect1	20,389,501	46.74%	90.22%	76.36%
infect2	20,927,458	45.68%	91.26%	74.36%
infect3	20,237,746	45.90%	90.70%	74.55%

Clean reads: the number of pair-end reads in clean date.

GC Content: the content of GC in clean date.

%≥Q30: The proportion of bases with Q≥30 in Clean Data.

Mapped reads: The proportion of Clean Reads mapped to the reference genome

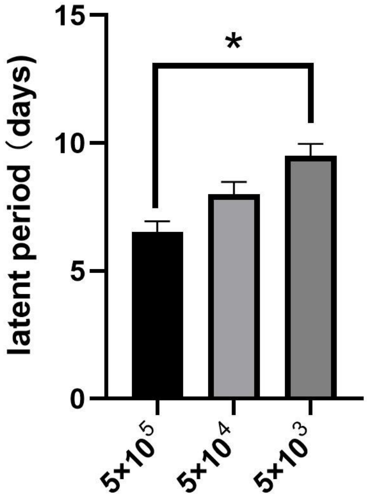

Figure S1. Latent period in *S. frugiperda* infected by *Nosema* sp. One-way ANOVA analysis and Tukey's multiple comparisons, $p<0.05$.

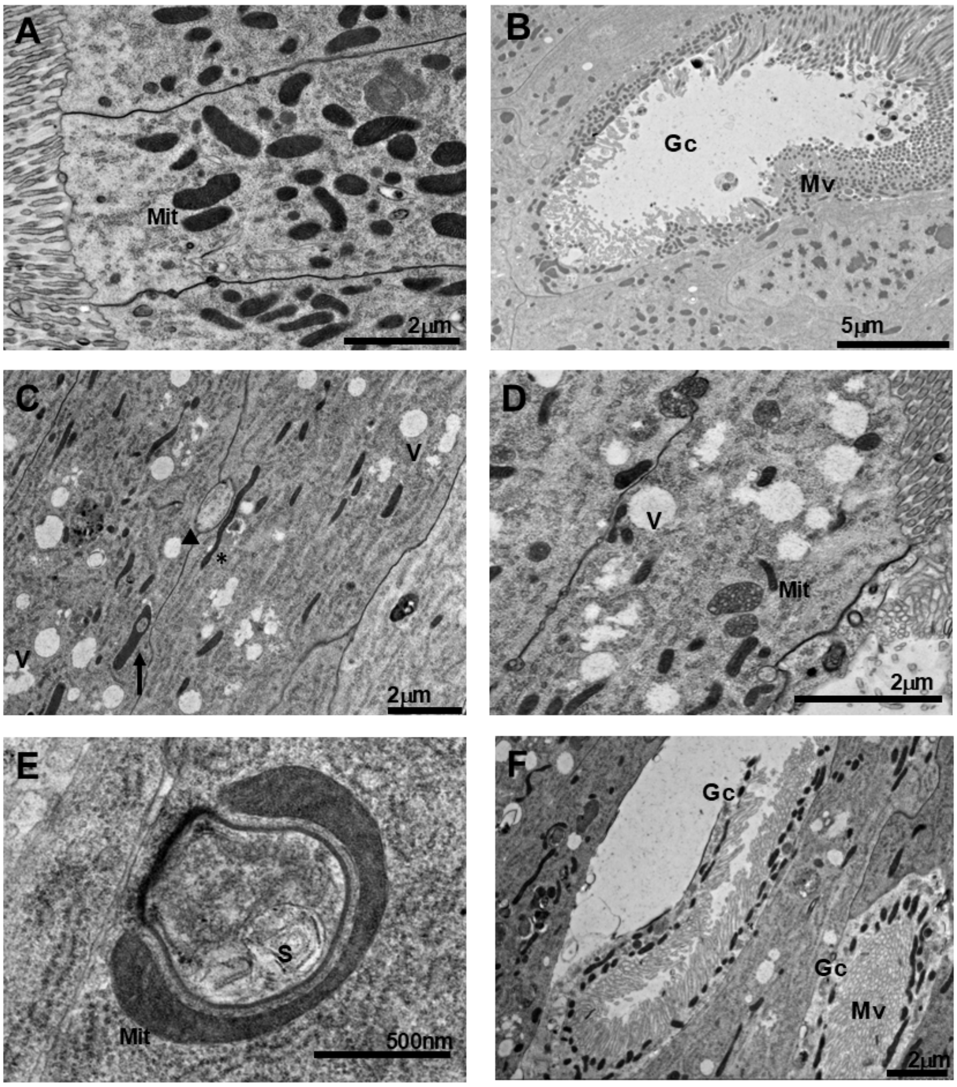

Figure S2. Ultra-histopathology of the midgut of *S. frugiperda* infected by *Nosema* sp. A-B: uninfected midgut; C-D: vacuole in midgut infected by *Nosema sp.* SF, arrow indicate vacuole appear in mitochondria, asterisk indicate mitochondria get thinner and longer, triangle indicate vacuole appear in Intercellular space. E: the binding of microsporidia to mitochondria. F: The goblet cells slough off, and the microvilli break, filling the goblet cavities. Mit: mitochondria; Gc: goblet cell; Mv: microvilli; V: vacuole; S: microsporidia.
